# 3′RNA and whole‐genome sequencing of archival uterine leiomyomas reveal a tumor subtype with chromosomal rearrangements affecting either *HMGA2*, *HMGA1*, or *PLAG1*

**DOI:** 10.1002/gcc.23088

**Published:** 2022-08-01

**Authors:** Vilja Jokinen, Miika Mehine, Siiri Reinikka, Sara Khamaiseh, Terhi Ahvenainen, Anna Äyräväinen, Päivi Härkki, Ralf Bützow, Annukka Pasanen, Pia Vahteristo

**Affiliations:** ^1^ Applied Tumor Genomics Research Program University of Helsinki Helsinki Finland; ^2^ Department of Medical and Clinical Genetics University of Helsinki Helsinki Finland; ^3^ iCAN Digital Precision Cancer Medicine Flagship Helsinki Finland; ^4^ Department of Obstetrics and Gynecology University of Helsinki and Helsinki University Hospital Helsinki Finland; ^5^ Department of Pathology University of Helsinki and HUSLAB, Helsinki University Hospital Helsinki Finland

**Keywords:** 3′RNA‐sequencing, chromosomal rearrangement, formalin‐fixed paraffin‐embedded tissue, uterine fibroid, uterine leiomyoma, whole‐genome sequencing

## Abstract

Uterine leiomyomas, or fibroids, are very common smooth muscle tumors that arise from the myometrium. They can be divided into distinct molecular subtypes. We have previously shown that 3′RNA‐sequencing is highly effective in classifying archival formalin‐fixed paraffin‐embedded (FFPE) leiomyomas according to the underlying mutation. In this study, we performed 3′RNA‐sequencing with 111 FFPE leiomyomas previously classified as negative for driver alterations in mediator complex subunit 12 (*MED12*), high mobility group AT‐hook 2 (HMGA2), and fumarate hydratase (FH) by Sanger sequencing and immunohistochemistry. This revealed 43 tumors that displayed expression features typically seen in HMGA2*‐*positive tumors, including overexpression of *PLAG1*. We explored 12 such leiomyomas by whole‐genome sequencing to identify their underlying genomic drivers and to evaluate the feasibility of detecting chromosomal driver alterations from FFPE material. Four tumors with significant HMGA2 overexpression at the protein‐level served as controls. We identified chromosomal rearrangements targeting either *HMGA2*, *HMGA1*, or *PLAG1* in all 16 tumors, demonstrating that it is possible to detect chromosomal driver alterations in archival leiomyoma specimens as old as 18 years. Furthermore, two tumors displayed biallelic loss of *DEPDC5* and one tumor harbored a *COL4A5–COL4A6* deletion. These observations suggest that instead of only HMGA2‐positive leiomyomas, a distinct leiomyoma subtype is characterized by rearrangements targeting either *HMGA2*, *HMGA1*, or *PLAG1.* The results indicate that the frequency of HMGA2‐positive leiomyomas may be higher than estimated in previous studies where immunohistochemistry has been used. This study also demonstrates the feasibility of detecting chromosomal driver alterations from archival FFPE material.

## 1 INTRODUCTION

Uterine leiomyomas, or fibroids, are common benign smooth muscle tumors. Their prevalence is 70% in reproductive age women.^1^ Leiomyomas are often asymptomatic, but every fourth patient suffers from symptoms like menorrhagia, pelvic pressure, urinary frequency, or constipation.^2^ Uterine leiomyomas may also affect fertility.^3^ Leiomyomas are the leading indication for hysterectomy worldwide and they pose a significant socio‐economic impact.^4^

A hotspot mutation in *MED12* or a chromosomal rearrangement of *HMGA2* that leads to a significant HMGA2 overexpression account for 80%–90% of all leiomyomas.^5^ Biallelic loss of *FH* constitutes a third subtype, which explains about 1% of uterine leiomyomas.^6^, ^7^, ^8^ Approximately 10% of uterine leiomyomas do not harbor mutations in any of these genes. Mutations in genes encoding for members of the SRCAP histone‐loading complex were recently discovered as a fourth rare molecular subtype.^9^ Such mutations were described in ~2% of leiomyomas.

Other less frequent aberrations have been reported in leiomyomas, but most of these have been detected as subclonal alterations that co‐occur with the established driver aberrations. In contrast to *HMGA2* rearrangements, high mobility group AT‐hook 1 (*HMGA1*) rearrangements are much rarer and may co‐occur with *MED12* mutations.^10^ We have previously proposed that *HMGA2* and *HMGA1* promote tumorigenesis by upregulating PLAG1 zinc finger (*PLAG1*).^11^ *PLAG1* rearrangements are common in other benign mesenchymal tumors, but their role in leiomyomas remains ambiguous.^12^ Leiomyomas with a collagen type IV alpha 5 chain (*COL4A5*) and collagen type IV alpha 6 chain (*COL4A6*) deletion resulting in overexpression of insulin receptor substrate 4 (*IRS4*) have been proposed as another leiomyoma subtype, but such aberrations have thus far been reported in only a small number of tumors.^11^, ^13^

Most high‐throughput sequencing studies on leiomyomas have been performed using fresh frozen tissue samples. Formalin‐fixed paraffin‐embedded (FFPE) tumors are routinely stored in hospital archives and such material could be used for comprehensive retrospective studies. However, formalin fixation induces DNA fragmentation and sequencing artifacts due to deamination and crosslinking.^14^ Detection of chromosomal alterations from FFPE tumors is therefore challenging. Nevertheless, we recently showed that archival FFPE leiomyomas can be effectively classified into established subtypes by 3′RNA‐sequencing.^15^ In this study, we performed 3′RNA‐sequencing to explore the expression pattern of 111 leiomyomas previously classified as negative for driver alterations in *MED12*, HMGA2, and FH by Sanger sequencing (*MED12*) and immunohistochemistry (HMGA2 and FH).^16^, ^17^, ^18^, ^19^, ^20^ These tumors are hereafter referred to as triple‐negative leiomyomas. Expression profiling revealed a subgroup of tumors that displayed features typical for HMGA2‐positive tumors. We analyzed such leiomyomas further by whole‐genome sequencing (WGS) to identify their genomic driver alterations and to evaluate the feasibility of WGS in detecting chromosomal alterations from archival FFPE material.

## 2 MATERIALS AND METHODS

### 2.1 Study material and sample selection

The research has been approved by the Ethics Review Board of the Hospital District of Helsinki and Uusimaa, Helsinki, Finland. All samples were collected with a signed informed consent from the patients or with authorization from the National Supervisory Authority for Welfare and Health (Valvira). The study material consisted of archival FFPE tissue samples and corresponding hematoxylin–eosin‐stained slides that were obtained from the Department of Pathology, Helsinki University Hospital, Helsinki, Finland. Histopathological evaluation of tumor and normal tissue slides was performed by a pathologist (Ralf Bützow or Annukka Pasanen). The status of *MED12*, HMGA2, and FH have been previously determined.^16^, ^17^, ^18^, ^19^, ^20^

### 2.2 RNA and DNA extraction

Total RNA was extracted and purified using the RNeasy® FFPE Kit (QIAGEN, Hilden, Germany) and the deparaffinization solution (QIAGEN) according to the manufacturer's protocol. The concentration and purity of the extracted RNA were analyzed using the LabChip GX Touch HT RNA Assay Reagent Kit (PerkinElmer, Waltham, MA) and the Qubit RNA BR kit (Thermo Fisher Scientific, Waltham, MA). Genomic DNA contamination was measured using the Qubit DNA BR kit (Thermo Fisher Scientific). DNA extraction was performed with the phenol‐chloroform method.

### 2.3 3′RNA‐sequencing

3′RNA‐sequencing of 111 leiomyoma samples was performed at Institute for Molecular Medicine Finland (FIMM) as previously described.^15^ In brief, FASTQ preprocessing was performed with default parameters using the QuantSeq 3′mRNA‐Seq Integrated Data Analysis Pipeline version 2.3.1 FWD UMI (Lexogen Gmbh, Vienna, Austria) implemented on the Bluebee® Genomics platform.^21^ Reads were trimmed using BBDuk, aligned against the Genome Reference Consortium human build 38 (GRCh38) using STAR, and counted using HTSeq.^22^, ^23^ Normalization, principal component analysis (PCA), and differential expression analysis were performed using DESeq2 implemented on the Chipster platform.^24^, ^25^ Supervised hierarchical clustering analysis was performed using the ComplexHeatmap v2.8.0 package.^26^ The clustering analysis was performed using the *HMGA1* gene and a set of six previously reported biomarkers for *HMGA2‐*positive leiomyomas.^11^ The data were grouped into five clusters using k‐means clustering.

### 2.4 Whole‐genome sequencing

WGS was performed with 16 leiomyomas and four myometrium samples (see Table S1 for an overview of the samples). Genomic libraries were prepared using the NEBNext Ultra II FS DNA Library Prep Kit No. E7805L (New England Biolabs, Ipswich, USA). Paired‐end WGS of 100 bp reads was performed at Beijing Genomics Institute (BGI) using the BGISEQ‐500 platform. FastQC was used to evaluate the quality of the sequencing reads and the data was preprocessed according to Genome Analysis Toolkit (GATK) v4 best practices guidelines.^27^, ^28^ The reads were trimmed by Trimmomatic v0.39 and aligned against the GRCh38 assembly by Burrows–Wheeler Aligner v0.7.17.^29^, ^30^ Duplicate reads were marked using GATK MarkDuplicates and recalibrated using BaseRecalibrator and ApplyBQSR.^28^ The quality of the aligned data was assessed by AlfredQC.^31^

Somatic chromosomal rearrangements were called by Delly v0.8.3 with the following parameters: −q 40, −s 20, and −c 150.^32^ Telomeres, centromeres, and alternate contigs were excluded using Delly's exclude list. All calls within or at most 75 000 bp upstream or downstream of the start and end positions of *HMGA2*, *HMGA1*, and *PLAG1* were carefully evaluated. Calls outside of these regions were filtered against the four myometrium and two in‐house blood samples with the following parameters: −a 0.1, −v 10, −m 500, −c 0.05, and −f somatic. Deletions and insertions shorter than 500 bp were removed and only calls with QUAL ≥ 300 were evaluated further. Identical calls in two or more tumors were considered false positives or normal variation. Delly filtered out some true calls due to the presence of false positive discordant reads in the same position in a control sample. Such calls were returned if QUAL ≥ 300 and the log_10_‐scale likelihood of the control sample being wildtype was ≥ −5. For some variant calls, Delly genotyped the tumor call as homozygous for the reference allele and thus filtered them out. These calls were returned if QUAL ≥ 300. All calls were visually evaluated using Integrative Genomics Viewer (IGV).^33^ Calls were excluded if they were located in regions containing many reads with poor mapping quality or if a similar set of discordant read pairs were also found in any of the four myometrium samples. For one sample (1250_1_S1) with high duplicate fraction and short insert size, the reads were trimmed to 75 bp for detecting chromosomal rearrangements, and the calls were filtered like in other samples, but QUAL > 300 was used instead of QUAL ≥ 300.

Somatic copy number alterations (SCNA) were detected using CNVkit v0.9.6 with default parameters.^34^ SCNA data were generated against a pooled normal reference generated with the four myometrium samples. Known problematic regions within the human genome were excluded using ENCODE blacklist.^35^

Mutect2 was used with the default parameters to call somatic point mutations and indels.^28^ Variant calls with a coverage of over 20 and an allelic fraction higher than 0.25 were filtered against variants found in a panel of normals (PON). PON was generated with samples from the 1000 Genomes Project, Genome Aggregation Database v2.1 and v3 (gnomAD), and an in‐house PON generated with 48 exomes and 28 genomes.^36^, ^37^ Variants were annotated with Ensembl Variant Effect Predictor and OncoKB.^38^, ^39^ Ensembl canonical reference transcripts were used unless stated otherwise.^40^ To visualize genetic alterations, IGV karyoploteR v1.18.0, RCircos v1.2.1, and FinchTV v1.4.0 were used.^33^, ^41^, ^42^, ^43^

### 2.5 Sanger sequencing validation

For each sample, one chromosomal rearrangement was selected for Sanger sequencing validation (Table S2). One consensus splice‐site mutation, one frameshift deletion in *DEPDC5*, and one *COL4A5–COL4A6* deletion were also validated by Sanger sequencing. Primer3Plus was used to design the primers.^44^ PCR was performed using DreamTaq DNA Polymerase (Thermo Fisher Scientific). The Sanger sequencing was conducted at the Sequencing Unit of the FIMM Technology Centre, Helsinki, and the electropherograms were analyzed using FinchTV v1.4.0.^43^

## 3 RESULTS

### 3.1 3′RNA‐sequencing identifies expression of *HMGA2* ‐subtype‐associated biomarkers in some leiomyomas previously classified as triple‐negative

**FIGURE 1:**
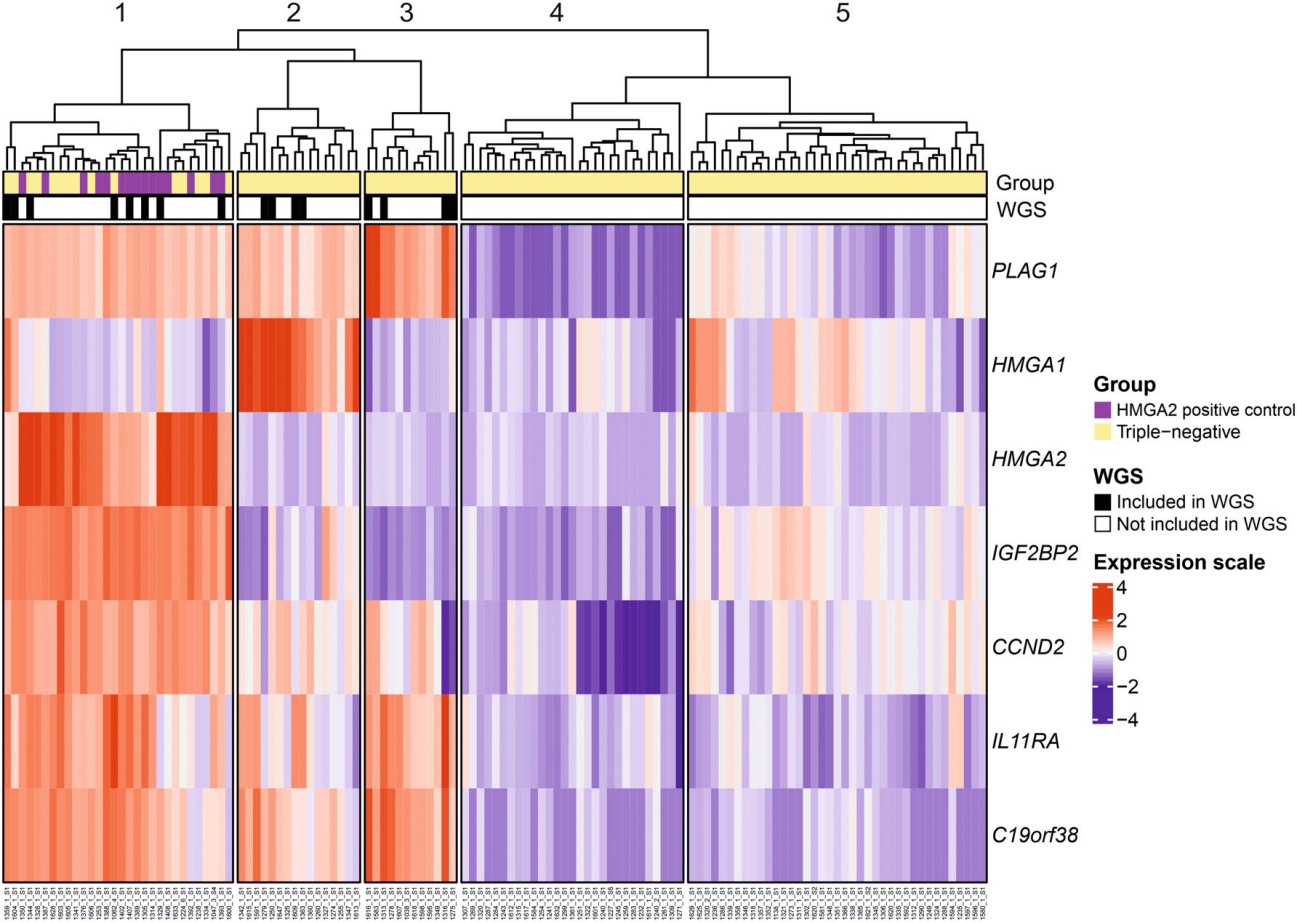
Supervised hierarchical clustering of 3′RNA‐sequencing data from 111 triple‐negative uterine leiomyomas and 15 HMGA2‐positive controls. Supervised hierarchical clustering analysis (k‐means clustering) was performed using the expression levels of *HMGA1* and six biomarkers for *HMGA2‐*positive leiomyomas (*HMGA2*, *IGF2BP2*, *CCND2*, *IL11RA*, *C19orf38*, and *PLAG1*). This divided the leiomyomas into two main branches. One of these comprised 58 tumors that displayed features typical for *HMGA2*‐positive tumors, including overexpression of *PLAG1*. The other branch comprised 68 samples without such characteristics. The *HMGA2*‐branch was subdivided further into three separate clusters: The first cluster was characterized by high expression of *HMGA2* (cluster 1), the second by high expression of *HMGA1* (cluster 2), and the third by exceptionally high expression of *PLAG1* but low expression of *HMGA2* and *HMGA1* (cluster 3). Based on the clustering analysis, we selected 16 leiomyomas for whole‐genome sequencing: eight with high expression of *HMGA2*, four with high expression of *HMGA1*, and four with high expression of *PLAG1* but low expression of *HMGA1* and *HMGA2*.

3′RNA‐sequencing of 111 leiomyomas that were previously classified as triple‐negative revealed high *HMGA1* or *HMGA2* expression in a subset of leiomyomas. Most of these samples also showed overexpression of *PLAG1*. To study this further, we performed supervised hierarchical clustering analysis using the *HMGA1* gene and six previously highlighted biomarkers for *HMGA2*‐positive leiomyomas (*HMGA2*, *IGF2BP2*, *CCND2*, *IL11RA*, *C19orf38*, and *PLAG1*).^11^ The triple‐negative samples were analyzed together with a previously published dataset of 15 HMGA2 overexpressing leiomyomas that served as positive controls. Of the 111 triple‐negative samples, 15 clustered together with the *HMGA2*‐positive controls (Figure 1). All these 15 tumors were originally classified as HMGA2‐negative by immunohistochemistry (eight samples with weak and seven with no expression of HMGA2). In addition to the *HMGA2* overexpressing tumors, we identified two other sets of tumors that clustered into the same branch with the *HMGA2*‐positive tumors. One of these clusters consisted of 16 leiomyomas that showed high expression of *HMGA1* and the other consisted of 12 leiomyomas that displayed exceptionally high expression of *PLAG1*, but low expression of both *HMGA1* and *HMGA2*.

Based on expression profiling, we selected 16 leiomyomas for WGS. First, we chose four samples that showed high *HMGA2* expression both at the 3′RNA and protein levels. These samples served as positive controls and were used to evaluate the feasibility of WGS in detecting chromosomal *HMGA2* alterations from archival FFPE samples. Second, we included 12 tumors that were previously classified as triple‐negative: four tumors that showed high *HMGA2* expression by 3′RNA‐sequencing but not at the protein level, four tumors with high *HMGA1* expression, and four tumors with high *PLAG1* but low *HMGA1* and *HMGA2* expression.

We then explored the global gene expression pattern of the 12 triple‐negative leiomyomas chosen for WGS. The samples were analyzed together with a previously published dataset of 44 leiomyomas with a driver alteration in *MED12*, *HMGA2*, or *FH* as well as five myometrium samples. The four HMGA2‐positive controls chosen for WGS were also included in this previously published dataset. PCA using the total transcriptome confirmed that all 16 samples chosen for WGS clustered among or close to the *HMGA2*‐positive tumors (Figure S1). These 16 samples are hereafter referred to as *HMGA‐*subtype leiomyomas. Most tumors in the *HMGA*‐group displayed cellular or conventional histology (Table S1).

### 3.2 WGS of *HMGA*‐subtype leiomyomas identifies recurrent rearrangements targeting either *HMGA2*, *HMGA1*, or *PLAG1*

WGS of 16 *HMGA‐*subtype leiomyomas revealed a candidate driver rearrangement in each tumor (Table 1). The detection of the rearrangements succeeded regardless of the sample age, which ranged from four to 18 years. In line with the 3′RNA‐sequencing results, the structural variant analysis detected an *HMGA2* rearrangement in eight leiomyomas, an *HMGA1* rearrangement in four leiomyomas, and a *PLAG1* rearrangement in four leiomyomas. All alterations were mutually exclusive. The rearrangements ranged from simple balanced translocations to more complex chromosomal rearrangements resembling chromothripsis. The chromothripsis‐like events were characterized by clustered breakpoints with alternating patterns of retention and loss that affected one to four chromosomes. Somatic copy number analysis identified recurrent deletions on Chromosomes 1, 14, 16, and 22 and recurrent amplifications on Chromosome 8 (Figure S2). The somatic copy number data supported the chromothripsis‐like events identified through structural variant analysis.

**TABLE 1:** Whole‐genome sequencing of 16 *HMGA*‐subtype leiomyomas revealed mutually exclusive rearrangements targeting either *HMGA2*, *HMGA1*, or *PLAG1*

Sample ID	Sample age (years)	HMGA2 immunohistochemistry	Expression, 3′RNA‐seq	Driver gene	Candidate partner gene	Relevant chromosomes involved	Type of alteration
1329_1_S1	8	++	High *HMGA2*	*HMGA2*	*RAD51B*	12, 14	Simple translocation
1393_1_S1	9	++	High *HMGA2*	*HMGA2*	*RAD51B*	11, 12, 14	Simple rearrangements
1407_1_S1	9	++	High *HMGA2*	*HMGA2*	*PTGER3* (downstream region)	1, 12	Balanced translocation
1305_1_S1	15	++	High *HMGA2*	*HMGA2*	unclear	12	Chromothripsis‐like
1344_1_S1	5	−	High *HMGA2*	*HMGA2*	*RAD51B*	12, 14	Balanced translocation
1062_4_S1	5	+	High *HMGA2*	*HMGA2*	*RAD51B*	12,14	Complex rearrangements
1604_1_S1	10	−	High *HMGA2*	*HMGA2*	Unclear / *RASSF3* (upstream region)	1, 3, 12, X	Chromothripsis‐like
1359_1_S1	4	+	High *HMGA2*	*HMGA2*	*PTGER3* (downstream region)	1, 12	Balanced translocation
1250_1_S1	16	−	High *HMGA1*	*HMGA1*	*RAD51B*	6, 14	Complex rearrangements
1279_1_S1	17	−	High *HMGA1*	*HMGA1*	*RAD51B*	6, 14	Chromothripsis‐like
1363_1_S1	4	−	High *HMGA1*	*HMGA1*	*PRDM1 / TRAF3IP2*	6, 15	Chromothripsis‐like
1609_1_S1	8	−	High *HMGA1*	*HMGA1*	*PBX1*	1, 6	Simple rearrangements
1275_1_S1	18	−	High *PLAG1*	*PLAG1*	*RBPMS*	8	Simple inversion
1616_1_S1	9	−	High *PLAG1*	*PLAG1*	*ACTG2*	2, 8, 13, X	Chromothripsis‐like
1316_1_S1	10	−	High *PLAG1*	*PLAG1*	*RNF19A*	2, 5, 8	Complex rearrangements
1313_1_S1	12	−	High *PLAG1*	*PLAG1*	*RBPJ*	4, 8	Simple rearrangements

### 3.3 *HMGA2* rearrangements

WGS revealed one or more chromosomal rearrangements targeting *HMGA2* (12q14.3) in all eight samples that displayed high *HMGA2* expression by 3′RNA‐sequencing (Table 1). We found no differences in the type of chromosomal rearrangements or in the translocation partners between the four positive controls with strong HMGA2 staining at the protein level and the four samples with weak or negative immunohistochemical staining.

Among the eight samples with an *HMGA2* rearrangement, breakpoints were detected upstream, downstream, or within *HMGA2*. Many samples displayed both adjacent and intragenic breakpoints (Figure S3A). The most common translocation partner for *HMGA2* was *RAD51B*, which was involved in four samples (Figure S4A). Two samples (1329_1_S1 and 1344_1_S1) harbored a characteristic translocation that combined full‐length *HMGA2* to the 5′ end of *RAD51B*, whereas two other samples (1393_1_S1 and 1062_4_S1) displayed more complex rearrangements (Figure S5).

**FIGURE 2:**
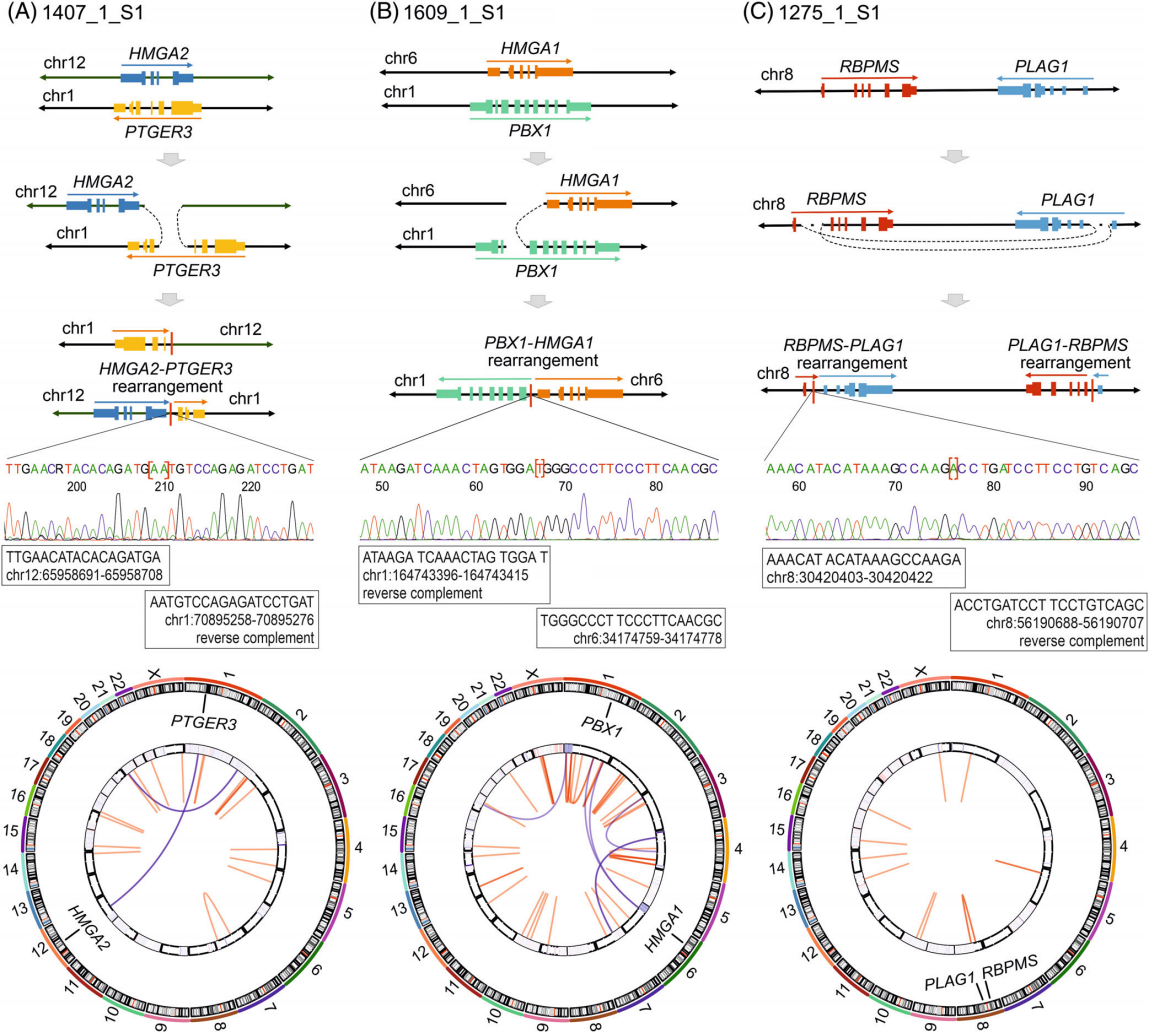
Whole‐genome sequencing of archival uterine leiomyomas identified chromosomal rearrangements targeting *HMGA2*, *HMGA1*, or *PLAG1*. We detected simple rearrangements targeting (A) *HMGA2* and *PTGER3* in sample 1407_1_S1, (B) *HMGA1* and *PBX1* in sample 1609_1_S1, and (C) *PLAG1* and *RBPMS* in sample 1275_1_S1. We validated one relevant rearrangement in each sample by Sanger sequencing. The schematic figures are not in scale, and only the most relevant breakpoints are shown. In the circos plots, intrachromosomal rearrangements are shown with red lines, interchromosomal rearrangements with blue lines, and copy number data by a heatmap in the inner circle.

Two samples displayed a rearrangement between *HMGA2* and Chromosome 1p31.1 where the prostaglandin E receptor 3 (*PTGER3*) is located (Figure S4B). One sample (1407_1_S1) harbored a rearrangement with breakpoints downstream of *HMGA2* and within intron 3 of *PTGER3* that combined full‐length *HMGA2* to the 3′ end of *PTGER3* (Figure 2A and Figure S6). The other sample (1359_1_S1) harbored breakpoints in intron 3 of *HMGA2* and downstream of *PTGER3*. This rearrangement combined the 5′ end of *HMGA2* to a region downstream of *PTGER3* (Figure S6).

**FIGURE 3:**
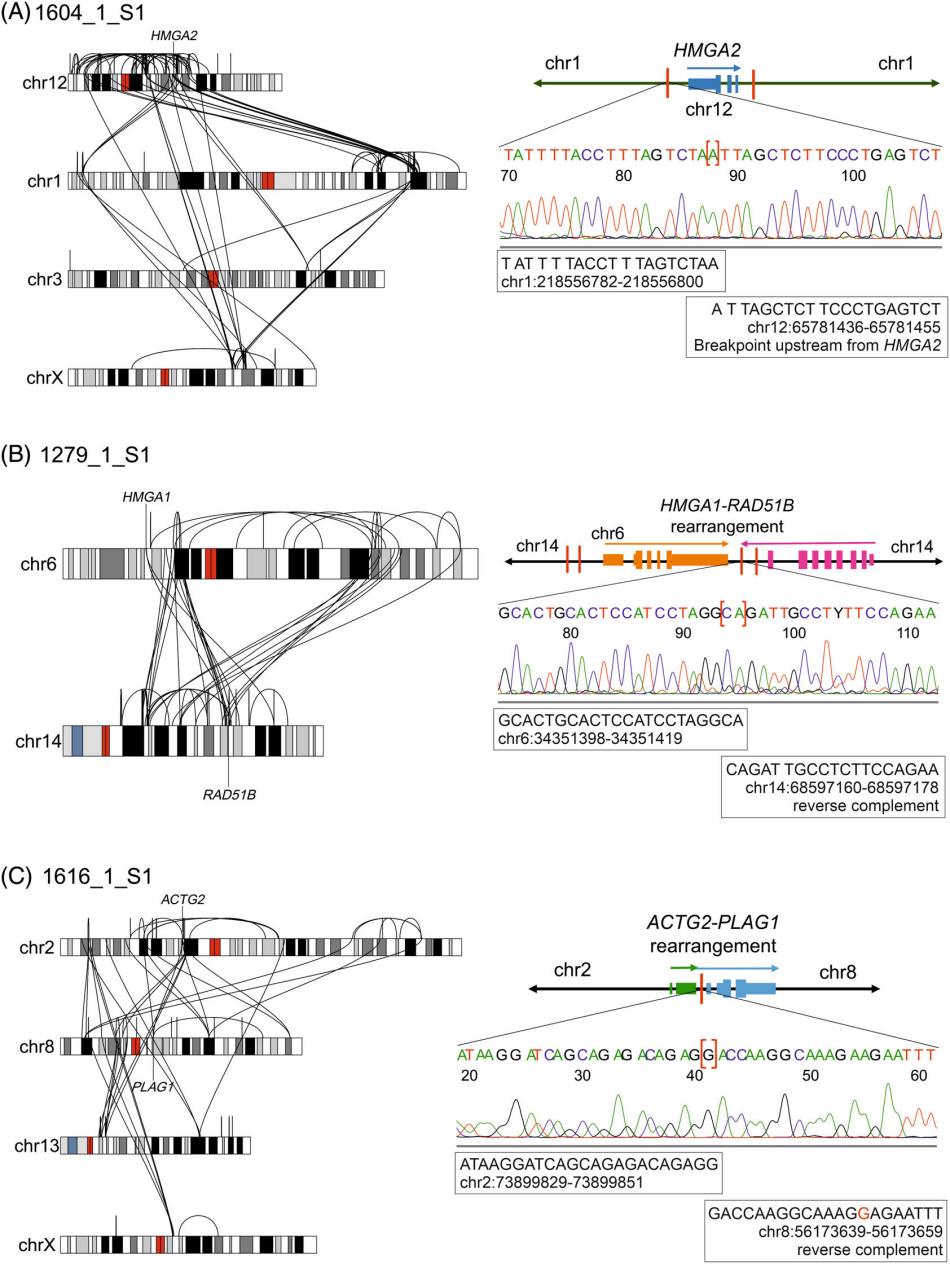
Whole‐genome sequencing of archival uterine leiomyomas revealed complex chromosomal rearrangements targeting *HMGA2*, *HMGA1*, or *PLAG1*. (A) We identified a chromothripsis‐like event, involving Chromosomes 1, 3, 12 (*HMGA2*), and X, in sample 1604_1_S1. We identified no clear translocation partner for *HMGA2*. We validated one rearrangement, involving a breakpoint upstream of *HMGA2* and a breakpoint in Chromosome 1, by Sanger sequencing. (B) We identified a complex chromosomal rearrangement between Chromosomes 6 (*HMGA1*) and 14 that juxtaposed *HMGA1* with the 5′ end of *RAD51B* in sample 1279_1_S1. We validated one of these rearrangements by Sanger sequencing. (C) We identified complex rearrangements involving Chromosomes 2, 8 (*PLAG1*), 13, and X in sample 1616_1_S1. We validated one rearrangement that combined the 5′end of *ACTG2* to the 3′ end of *PLAG1* by Sanger sequencing. The schematic figures are not in scale, and only the most relevant breakpoints are shown.

We detected a chromothripsis‐like event involving *HMGA2* in two samples. In sample 1305_1_S1, we detected a mild chromothripsis‐like event affecting Chromosome 12. This resulted in breakpoints both upstream of and within *HMGA2* (Figure S7). Intron 3 contained a rearrangement that combined the 5′ end of *HMGA2* to 12p11.22, but we could not identify a candidate partner gene in this region. The upstream rearrangement combined *HMGA2* to a region upstream of *RASSF3*. The other sample (1604_1_S1) harbored a chromothripsis‐like event involving Chromosomes 1, 3, 12, and X (Figure 3A and Figure S7). This resulted in breakpoints upstream, downstream, and within intron 3 of *HMGA2*. We did not identify a candidate partner gene for *HMGA2* in this sample.

### 3.4 *HMGA1* rearrangements

We detected an *HMGA1* (6p21.31) rearrangement in all four samples with high *HMGA1* expression (Table 1). Two samples displayed upstream breakpoints, one sample displayed downstream breakpoints, and one sample displayed both upstream and downstream breakpoints of *HMGA1* (Figure S3B). Sample 1609_1_S1 harbored a rearrangement between Chromosomes 6p21.31 and 1q23.3 with a breakpoint upstream of *HMGA1* and a breakpoint in intron 2 of *PBX1*. This alteration combined full‐length *HMGA1* to the 3′ end of *PBX1* (Figure 2B and Figure S8). One sample (1363_1_S1) harbored several intrachromosomal rearrangements on Chromosome 6 and interchromosomal rearrangements between Chromosomes 6 and 15. This chromothripsis‐like event resulted in breakpoints upstream of *HMGA1*, upstream of and within intron 1 of *PRDM1* (transcript ENST00000651185.1), and in intron 6 of *TRAF3IP2*. This complex event combined full‐length *HMGA1* to the 3′ end of *TRAF3IP2* with exon 1 of *PRDM1* located in between (Figure S8). Sample 1279_1_S1 displayed a chromothripsis‐like event involving Chromosomes 14 and 6 with breakpoints both upstream and downstream of *HMGA1* (Figure 3B and Figure S8). This resulted in a rearrangement that included breakpoints downstream of *HMGA1* and in intron 10 of *RAD51B* and the event combined full‐length *HMGA1* to the 5′ end of *RAD51B*. Initially, we did not detect any rearrangements affecting *HMGA1*, *HMGA2*, or *PLAG1* in sample 1250_1_S1 that displayed poor sequencing data quality (Table S1). After trimming the reads from 100 to 75 bp, we detected a rearrangement with breakpoints downstream of *HMGA1* and downstream of *RAD51B* (Figure S8).

### 3.5 *PLAG1* rearrangements

We identified a *PLAG1* (8q12.1) rearrangement in all four samples that showed high *PLAG1* expression but low *HMGA1* and *HMGA2* expression. Each sample harbored intronic breakpoints that combined the 3′ end of *PLAG1* to the 5′ end of another gene (Figure S3C). One sample (1313_1_S1) harbored a rearrangement between Chromosomes 8q12.1 and 4p15.2 with intragenic breakpoints in intron 1 of *PLAG1* and intron 2 of *RBPJ* (Figure S9). Another sample (1316_1_S1) harbored multiple rearrangements between Chromosomes 8 and 2, including intragenic breakpoints in intron 1 of *PLAG1* and in intron 1 of *RNF19A* (Figure S9). The third sample (1275_1_S1) displayed a simple inversion inv (8)(p12q12.1) with intragenic breakpoints in intron 1 of *PLAG1* and intron 1 of *RBPMS* (Figure 2C and Figure S9). The fourth sample (1616_1_S1) harbored a chromothripsis‐like event involving Chromosomes 2, 8, 13, and X (Figure 3C and Figure S9). This event included a rearrangement between Chromosomes 8q12.1 and 2p13.1 with intragenic breakpoints in intron 2 of *PLAG1* and intron 2 of *ACTG2*.

### 3.6 Biallelic loss of *DEPDC5* and a  *COL4A5‐COL4A6* deletion as likely secondary driver events

**FIGURE 4:**
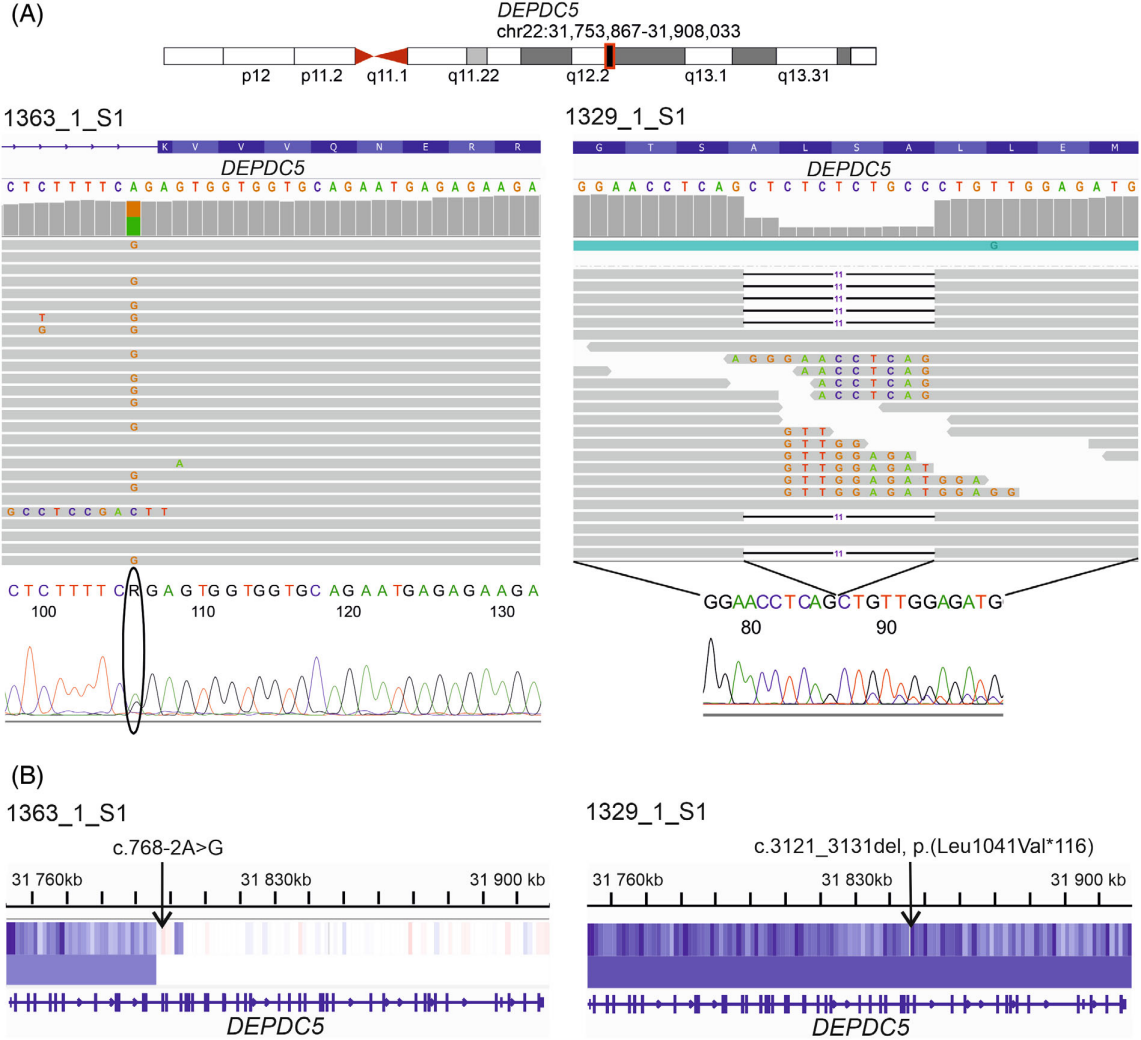
Biallelic loss of *DEPDC5* in two *HMGA*‐subtype uterine leiomyomas. (A) We identified a consensus splice site substitution c.768‐2A > G in *DEPDC5* in one sample with an *HMGA1* rearrangement and a frameshift mutation c.3121_3131del, p.(Leu1041Val*116) in *DEPDC5* in one sample with an *HMGA2* rearrangement. (B) Somatic copy number data revealed that both mutations were accompanied by a larger deletion on Chromosome 22 resulting in biallelic loss of *DEPDC5*.

**FIGURE 5:**
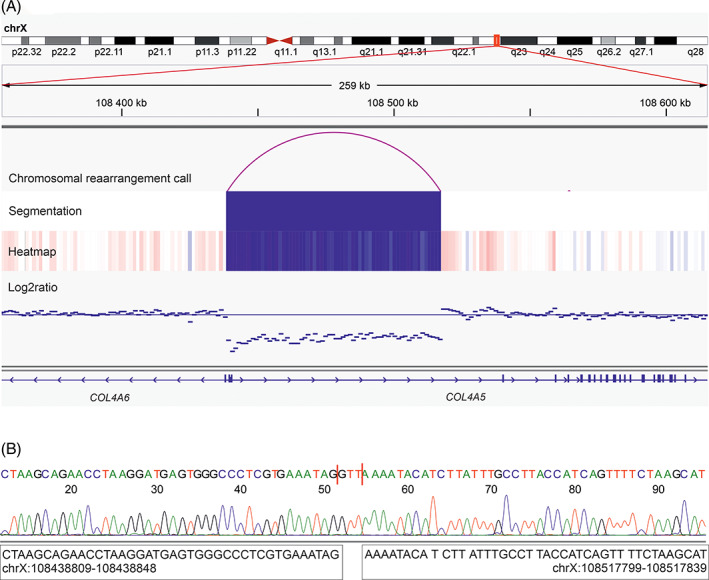
*COL4A5–COL4A6* deletion in one *HMGA*‐subtype uterine leiomyoma. (A) Whole‐genome sequencing revealed a deletion affecting the 5′ ends of *COL4A5* and *COL4A6* in sample 1250_1_S1 that also harbored an *HMGA1* rearrangement. (B) The deletion was validated by Sanger sequencing with breakpoints in intron 1 of *COL4A5* and intron 1 of *COL4A6*.

To identify point mutations and indels that might have contributed to the development of the 16 *HMGA*‐subtype leiomyomas, we explored the WGS data for recurrently mutated genes and genes previously suggested as drivers in leiomyomas. We identified four protein‐coding genes that were recurrently mutated by a nonsynonymous point mutation or a microindel (Table S3). DEP domain containing 5 *(DEPDC5*) was the only recurrently mutated gene that harbored pathogenic or likely pathogenic mutations according to VarSome.^45^ Both samples with a gene‐level mutation in *DEPDC5* harbored a large deletion on Chromosome 22 as a second hit (Figure 4). In addition, one sample (1250_1_S1) harbored a deletion of 78 950 bp affecting the 5′ ends of *COL4A5* and *COL4A6* (Figure 5A and B). 3′RNA‐sequencing revealed a 27‐fold increase in *IRS4* expression in this sample compared with five myometrium controls. We detected no SRCAP complex mutations in any of the 16 tumors.

## 4 DISCUSSION

Uterine leiomyomas are benign smooth muscle tumors that can be classified into distinct molecular subtypes based on their mutational and gene expression profiles.^11^ We recently showed that 3′RNA‐sequencing accurately classifies archival FFPE leiomyomas with known driver alterations.^15^ Here, we performed 3′RNA‐sequencing with 111 leiomyomas that had been previously classified as negative for a *MED12* mutation, HMGA2 overexpression, and FH‐deficiency by Sanger sequencing or immunohistochemistry.^16^, ^17^, ^18^, ^19^, ^20^ Interestingly, we identified a subset of tumors to display expression features typically seen in leiomyomas with an *HMGA2* rearrangement, including *PLAG1* overexpression. Gene expression profiling divided the samples further into three separate clusters, one characterized by high expression of *HMGA2*, one by upregulation of *HMGA1*, and one by exceptionally high expression of *PLAG1* but low expression of both *HMGA2* and *HMGA1*. We then performed WGS to identify driver alterations in 16 such leiomyomas.

WGS identified an *HMGA2* rearrangement in all eight tumors that displayed *HMGA2* overexpression by 3′RNA‐sequencing, including the four leiomyomas without strong HMGA2 protein expression. All these four tumors showed at least some areas of weak HMGA2 expression by immunohistochemistry. Previously, only samples with strong protein expression have been classified as HMGA2‐positive, but the results of this study suggest that also some samples with weaker expression are likely to harbor *HMGA2* rearrangements. Our results indicate that the frequency of *HMGA2‐*positive leiomyomas may have been underestimated in previous studies where only immunohistochemistry has been used. Nevertheless, immunohistochemistry is a quick and inexpensive method that classifies most FFPE leiomyomas correctly.

In addition to *HMGA2* rearrangements, we identified an *HMGA1* rearrangement in tumors with high *HMGA1* expression and a *PLAG1* rearrangement in tumors with exceptionally high *PLAG1* expression. Unlike *HMGA2* rearrangements, *HMGA1* and *PLAG1* rearrangements are rare in leiomyomas and may co‐occur with *MED12* mutations, suggesting that they are secondary events related to tumor progression.^10^ However, none of the samples with an *HMGA1* or *PLAG1* rearrangement in this study harbored an alteration in a well‐established leiomyoma driver gene and all displayed expression features associated with leiomyomas of the *HMGA2‐*subtype. This indicates that *HMGA1* and *PLAG1* rearrangements may also act as initiators of tumorigenesis. These results also suggest that leiomyomas with an *HMGA2*, *HMGA1*, or *PLAG1* aberration form a distinct leiomyoma subtype with similar molecular downstream consequences in tumorigenesis.

*HMGA2* encodes a nuclear transcription factor characterized by three DNA‐binding domains known as AT‐hooks.^46^ HMGA2 indirectly regulates gene expression by binding to the minor groove of AT‐rich DNA sequences and thereby induces changes in chromatin structure.^47^ *HMGA1* encodes another chromatin regulating protein with a similar structure and functionality, suggesting that both genes regulate, at least in part, the same set of genes.^46^ *HMGA2* and *HMGA1* rearrangements have both been reported in multiple benign mesenchymal tumor types, including lipomas, endometrial polyps, and pulmonary chondroid hamartomas.^46^ HMGA2 and HMGA1 have been proposed to promote tumorigenesis through activation of *PLAG1*.^*11*^ *PLAG1* encodes for a zinc finger transcription factor that regulates the transcription of many genes, including insulin‐like growth factor 2 (*IGF2*).^48^ *PLAG1* is frequently upregulated by translocations in several benign mesenchymal tumors, including pleomorphic adenomas and lipoblastomas.^49^, ^50^ HMGA2 is widely used as a biomarker for leiomyomas of the *HMGA2*‐subtype, but our results indicate that *PLAG1* expression could serve as a biomarker for the whole *HMGA‐*subtype.

Unlike *HMGA1* rearrangements, some *HMGA2* and all *PLAG1* rearrangements involved intragenic breakpoints, suggesting that such rearrangements could result in oncogenic fusion proteins. However, the formation of fusion proteins is unlikely as the breakpoints were located in the first two introns of *PLAG1*, and the first three exons contain only 5′UTR. Thus, all the coding exons and the initiation codon of *PLAG1* remained intact in each rearrangement. Leiomyomas with an intragenic breakpoint in *HMGA2* were also not predicted to form fusion genes based on the orientation of some of the rearrangement partners. These results indicate that instead of fusion genes, promoter hijacking leading to upregulation is the principal mechanism by which these three genes promote tumorigenesis.

Early cytogenetic studies on leiomyomas and other benign mesenchymal tumors identified *RAD51B* as the most common translocation partner for *HMGA2*, often in the form of a balanced translocation.^51^ WGS has since shown that a subset of leiomyomas harbor highly complex chromosomal rearrangements involving a variety of translocation partners.^13^ Such chromothripsis‐like rearrangements are characterized by several clustered and interconnected breakpoints affecting one or a few chromosomes. Loss of *RAD51B* has also been proposed to promote tumorigenesis, and its involvement as a translocation partner results in the strongest *HMGA2* overexpression compared with other translocation partners.^13^ In this study, we identified both simple translocations and chromothripsis‐like rearrangements involving *RAD51B* as a translocation partner for both *HMGA2* and *HMGA1*. *RAD51B* has also been reported as a translocation partner for *PLAG1* in lipoblastomas.^52^ This is the first study to report a downstream region of *PTGER3* as a recurrent rearrangement partner for *HMGA2*. *PTGER3* is highly expressed in the uterus and smooth muscle tissue.^53^, ^54^ In a few leiomyomas, we detected *HMGA2* or *HMGA1* to be combined with a candidate partner gene in opposite directions. Enhancer hijacking or loss of a repressor rather than promoter hijacking may explain the overexpression in such cases. A few leiomyomas displayed breakpoints downstream of *HMGA2* and *HMGA1*, suggesting that also downstream rearrangements could result in their upregulation. In each sample with a *PLAG1* rearrangement, the 3′ end of *PLAG1*, including all the coding exons, was combined with the 5′ end of another gene. All the identified candidate partner genes, *ACTG2*, *RBPJ*, *RNF19A*, and *RBPMS*, are highly expressed in the uterus under normal conditions, indicating that *PLAG1* rearrangements result in promoter hijacking.^53^, ^54^

In addition to rearrangements of *HMGA1*, *HMGA2*, and *PLAG1*, we identified biallelic loss of *DEPDC5* in one leiomyoma with an *HMGA2* rearrangement and in another leiomyoma with an *HMGA1* rearrangement. We have previously reported biallelic loss of *DEPDC5* as a secondary event in four clonally related leiomyomas from one patient and in one leiomyoma from another patient.^55^ All five tumors harbored an *HMGA2* rearrangement as well, suggesting that *DEPDC5* is involved in tumor progression rather than initiation. Subsequently, biallelic loss of *DEPDC5* has been found to be common in gastrointestinal stromal tumors with reduced sensitivity to KIT inhibitors, indicating that such alterations can also act as secondary events related to drug resistance.^56^ DEPDC5 is a subunit of the GATOR1 complex, which functions as an inhibitor of the mTORC1 signaling pathway.^57^

Finally, we identified one sample with a *COL4A5–COL4A6* deletion and significant upregulation of *IRS4*. We have previously proposed that these deletions could represent a distinct leiomyoma subtype, but such aberrations have thus far been reported in only a small number of leiomyomas.^11^, ^13^ This sample harbored also an *HMGA1* rearrangement and the tumor displayed an *HMGA*‐type expression profile, suggesting that the *COL4A5–COL4A6* deletion is a secondary event. To the best of our knowledge, this is the first time when a *COL4A5–COL4A6* deletion has been identified in a leiomyoma that also harbors another driver mutation.

The sample series in this study included archival tissue specimens as old as 18 years, demonstrating that it is possible to detect chromosomal driver alterations from archival FFPE material. The chromosomal rearrangements in our dataset were often complex involving multiple breakpoints and it is therefore unlikely that all relevant breakpoints were identified. Although we could not define the exact translocation partners for *HMGA2*, *HMGA1*, and *PLAG1* in all cases, at least one important rearrangement was validated by Sanger sequencing in each sample. The data presented in this study indicate that archival FFPE samples are particularly applicable for targeted sequencing, combined with preliminary information such as expression data from immunohistochemistry or RNA‐sequencing that can be utilized to define the target regions.

Aberrations in *MED12*, *HMGA2*, *FH*, and SRCAP complex genes account for most leiomyomas, but ~10% of leiomyomas do not harbor defects in any of these genes.^9^ The results of this study imply that *HMGA1* and *PLAG1* rearrangements are likely to explain a small proportion of uterine leiomyomas with no mutations in the well‐established driver genes.

To conclude, we have here confirmed that leiomyomas with an *HMGA2*, *HMGA1*, or *PLAG1* rearrangement share multiple molecular features, including similar gene expression patterns and shared translocation partners. This suggests that these tumors form a distinct leiomyoma subtype, expanding the *HMGA2‐*subtype to an *HMGA*‐subtype. Although some studies have indicated that *HMGA1* overexpressing leiomyomas result in a distinct expression profile,^58^ our observations are compatible with a recent RNA‐sequencing study on 276 leiomyomas in which most *HMGA2* and *HMGA1* overexpressing leiomyomas clustered together.^9^ *HMGA2* rearrangements have been associated with larger tumor size and a smaller number of tumors compared with leiomyomas with a *MED12* mutation.^20^ More data are needed to evaluate whether these clinical associations apply to the entire *HMGA*‐subtype. This study also highlights the feasibility of 3′RNA‐sequencing in classifying archival tissue material and confirms that both simple and complex chromosomal rearrangements can be detected from archival FFPE sample‐derived WGS data. Millions of women suffer from leiomyomas, and the ability to accurately stratify each lesion should pave the way towards personalized treatments. Cell line studies have already demonstrated that angiogenic factors play a role in HMGA2‐mediated tumorigenesis, indicating therapeutic potential of angiogenesis inhibitors in *HMGA2‐*positive leiomyomas.^59^ Further studies are warranted to evaluate their feasibility in clinical setting and to assess their applicability in *HMGA‐*subtype leiomyomas.

## AUTHOR CONTRIBUTIONS

Miike Mehine and Pia Vahteristo designed and supervised the study. Anna Äyräväinen and Päivi Härkki provided the samples. Siiri Reinikka, Sara Khamaiseh, and Terhi Ahvenainen extracted DNA, collected sample information, and coordinated the NGS analyses. Vilja Jokinen and Miika Mehine performed the NGS data analyses. Vilja Jokinen performed Sanger sequencing. Ralf Bützow and Annukka Pasanen evaluated tumor histopathology and scored immunohistochemical staining. Vilja Jokinen, Miika Mehine, and Pia Vahteristo wrote the article. All authors reviewed and approved the article.

## CONFLICT OF INTEREST

The authors declare that they have no conflict of interest.

## Supporting information


**Appendix S1** Supporting Information

## Data Availability

All key findings are presented in the manuscript and supplementary files. The raw data are not publicly available due to compliance with the ethics approval and confidentiality agreements. Data may be obtained from the authors upon reasonable request when compatible with European General Data Protection Regulation (GDPR) and with the permission from the University of Helsinki.
