# Supplementary material for: 3′RNA and whole‐genome sequencing of archival uterine leiomyomas reveal a tumor subtype with chromosomal rearrangements affecting either HMGA2, HMGA1, or PLAG1
Source: Genes Chromosomes Cancer. 2022 Aug 1;62(1):27–38. doi: 10.1002/gcc.23088 (PMC9804854; doi:10.1002/gcc.23088)
Supplement: Supplementary file 1 — Appendix S1 Supporting Information [file GCC-62-27-s001.docx]

# Supplementary Material

**Supplementary Table 1**. Sequencing coverage and quality of the whole-genome sequencing data from 16 *HMGA* subtype uterine leiomyoma and four myometrium FFPE samples.

| **Sample ID** | **Histopathology** | **Duplicate fraction** | **Median insert size** | **Median**  **coverage per base** | **Percentage of targeted bases with**  **coverage ≥10** |
| --- | --- | --- | --- | --- | --- |
| 1062_4_S1 | Cellular (Highly) | 0.0323 | 176 | 63 | 98.17 |
| 1250_1_S1 | Mitotically active | 0.0917 (0.0906) | 137 (129) | 57 (44) | 97.64 (96.78) |
| 1275_1_S1 | Cellular (Highly) | 0.0685 | 137 | 59 | 98.07 |
| 1279_1_S1 | Cellular (Highly) | 0.0546 | 145 | 61 | 97.86 |
| 1305_1_S1 | Cellular (Highly) | 0.0596 | 153 | 56 | 97.44 |
| 1316_1_S1 | Bizarre nuclei | 0.0604 | 155 | 52 | 96.53 |
| 1313_1_S1 | Cellular | 0.0521 | 138 | 63 | 98.31 |
| 1329_1_S1 | Cellular | 0.0538 | 157 | 64 | 97.94 |
| 1344_1_S1 | Cellular | 0.0439 | 171 | 67 | 98.19 |
| 1359_1_S1 | Cellular (Highly) | 0.0318 | 171 | 67 | 98.21 |
| 1363_1_S1 | Cellular | 0.0384 | 161 | 64 | 98.16 |
| 1393_1_S1 | Conventional | 0.0466 | 155 | 57 | 97.72 |
| 1407_1_S1 | Conventional | 0.0376 | 158 | 68 | 98.34 |
| 1604_1_S1 | Conventional | 0.0325 | 164 | 64 | 98.18 |
| 1609_1_S1 | Conventional | 0.0452 | 162 | 57 | 97.18 |
| 1616_1_S1 | Conventional | 0.0484 | 163 | 59 | 97.85 |
| 1574_1_S1 | Myometrium | 0.0614 | 153 | 34 | 94.96 |
| 1575_1_S1 | Myometrium | 0.1598 | 147 | 31 | 93.88 |
| 1576_1_S1 | Myometrium | 0.1649 | 144 | 22 | 86.76 |
| 1577_1_S1 | Myometrium | 0.0446 | 163 | 31 | 95.31 |

For sample 1250_1_S1, values in parentheses refer to data after trimming 100bp reads to 75bp.

**Supplementary Table 2**. Technical information of PCR reactions for Sanger sequencing validation of selected genetic alterations in leiomyoma samples.

| **Sample ID** | **Alteration** | **Primers** | **Annealing temperature (°C)** | **DMSO** |
| --- | --- | --- | --- | --- |
| 1329_1_S1 | *RAD51B-HMGA2* | F: tgcccctccctaggataact  R: ctaaagcaagggttggcaag | 58.0 | 5 % |
|  | *DEPDC5* frameshift deletion | F: tgatggccttgtctcctctt  R: ggggacaagtcagtcttcca | 59.0 | no |
| 1393_1_S1 | *RAD51B-HMGA2* | F: acaagcaaagaagccacagg  R: tgggcttatacaggcatatttca | 59.0 | no |
| 1407_1_S1 | *HMGA2-PTGER3* | F: accaggagcaagagcatagc  R: tcatgaacgtggaaaactcct | 56.0 | 5 % |
| 1305_1_S1 | Intrachromosomal rearrangement with the breakpoint within *HMGA2* | F: tggtgtggtgcatcataaagt  R: ggtacccatgcaaccattct | 58.0 | no |
| 1344_1_S1 | *RAD51B-HMGA2* | F: cgcgtgcctataatcctagc  R: ctggcactggttcccattat | 58.6 | no |
| 1062_4_S1 | *HMGA2-RAD51B* | F: taaagggaaggagcgagaca  R: cagcctttcatctcctctgc | 58.6 | no |
| 1604_1_S1 | Interchromosomal rearrangement with breakpoints upstream from *HMGA2* and in 1q41 | F: tcatccagactgttgctcgt  R: ttgaatccatgtgaatgacactt | 58.6 | no |
| 1359_1_S1 | Interchromosomal rearrangement combining *HMGA2* with a downstream region of *PTGER3* | F: aagggaagtcctttaggctga  R: ggcccataatggtgcttact | 58.6 | no |
| 1250_1_S1 | Interchromosomal rearrangement with breakpoints downstream of *HMGA1* and *RAD51B* | F: gcttgaaagctgtcaggaat  R: ctgggaatagcgcactatgag | 59.0 | no |
|  | *COL4A5-6* deletion | F: ccactctgagctaagcagaacc  R: cccaataaggacaataagagaaataaa | 59.0 | no |
| 1363_1_S1 | *PRDM1-HMGA1* | F: agggccaaaggctctatgat  R: ctgcccctttgtctggtg | 58.6 | no |
|  | *DEPDC5* splice site mutation | F: tccctcgacacatgggtatt  R: atgcagtacctgcctgttcc | 59.0 | no |
| 1279_1_S1 | *HMGA1-RAD51B* | F: tcctcttgtgcctgtcattg  R: agacctagtggctggctgag | 58.0 | 5 % |
| 1609_1_S1 | *PBX1-HMGA1* | F: gaaacacatcgcttccaaca  R: ggataccagcctagccacag | 58.6 | no |
| 1275_1_S1 | *RBPMS-PLAG1* | F: tcccaagtactttaaacagaatttgac  R: gctgaaggggaaggtacaga | 59.0 | no |
| 1616_1_S1 | *ACTG2-PLAG1* | F: ccttgtgggctggtttagag  R: tgaaaagtgcaacaaaacttgg | 58.6 | no |
| 1316_1_S1 | *RNF19A-PLAG1* | F: tggacctttactgggcactc | 59.0 | no |
|  |  | R: tcctcctacctcagcctcct |  |  |
| 1313_1_S1 | *RBPJ-PLAG1* | F: atccggtcacccttttcttt  R: aaaaagtccatgaagcaaactattc | 58.0 | no |

**Supplementary Table 3.** Point mutations and indels in genes mutated in more than one leiomyoma.

| **Gene** | **Sample ID** | **Variant type** | **Mutation** | **Predicted protein alteration** | **Consequence**  **(ACMG*, VarSome)** |
| --- | --- | --- | --- | --- | --- |
| *DEPDC5* | 1329_1_S1 | Frame-shift deletion | c.3121_3131del | p.(Leu1041Valfs*116) | Pathogenic |
|  | 1363_1_S1 | Splice acceptor variant | c.768-2A>G | - | Pathogenic |
| *LRIG2* | 1363_1_S1 | Missense mutation | c.533G>A | p.(Arg178Lys) | Uncertain Significance |
|  | 1609_1_S1 | Missense mutation | c.814C>G | p.(His272Asp) | Likely Benign |
| *PLB1* | 1316_1_S1 | Missense mutation | c.139C>T | p.(Pro47Ser) | Likely Benign |
|  | 1616_1_S1 | Missense mutation | c.773A>T | p.(Gln258Leu) | Likely Benign |
| *UNC5D* | 1062_4_S1 | Missense mutation | c.883T>C | p.(Ser295Pro) | Uncertain Significance |
|  | 1359_1_S1 | In-frame deletion | c.811_813del | p.(Gly271del) | Uncertain Significance with some pathogenic evidence |

*American College of Medical Genetics and Genomics classification of clinical significance


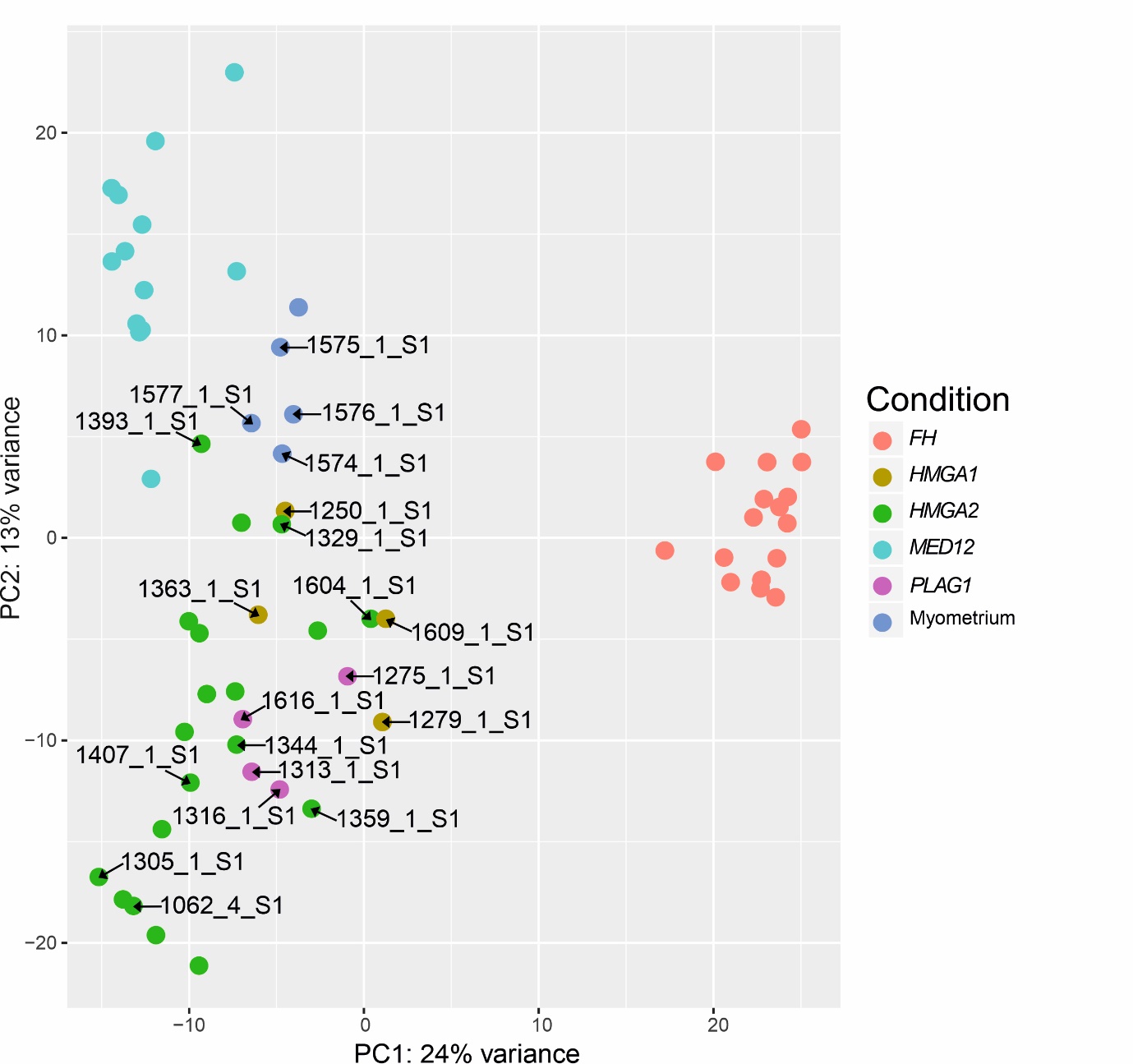
**Supplementary Figure 1. Principal component analysis using 56 uterine leiomyomas and five myometrium samples.** Principal component analysis using the total transcriptome was performed with 12 triple-negative leiomyomas and a previously published dataset of five myometrium samples and 44 leiomyomas with a known driver defect (13 leiomyomas with a *MED12* mutation, 15 HMGA2-positive by immunohistochemistry, and 16 with FH-deficiency). Whole-genome sequencing was performed with four HMGA2-positive control tumors, four myometrium samples, and 12 triple-negative leiomyomas (marked with sample identifiers). In addition to confirming an *HMGA2* rearrangement in the four HMGA2-positive controls, whole-genome sequencing identified a *PLAG1* rearrangement in four, an *HMGA1* rearrangement in four, and an *HMGA2* rearrangement in four triple-negative leiomyomas. All these 12 tumors clustered among or close to leiomyomas of the *HMGA2* subtype.


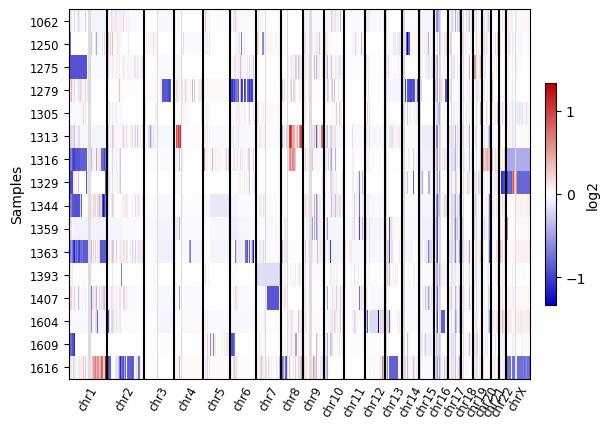
**Supplementary Figure 2. Copy number profiles generated from the whole-genome data of 16 *HMGA* subtype leiomyomas.** Somatic copy number analysis revealed recurrent large deletions on chromosome 1p in seven leiomyomas. Four of these samples harbored large deletions also on chromosome 1q. We identified loss of whole chromosome X in three leiomyomas, deletions of chromosome 2p in two leiomyomas, and deletions of chromosome 16q in two leiomyomas. The figure was plotted using CNVkit v0.9.6.^1^


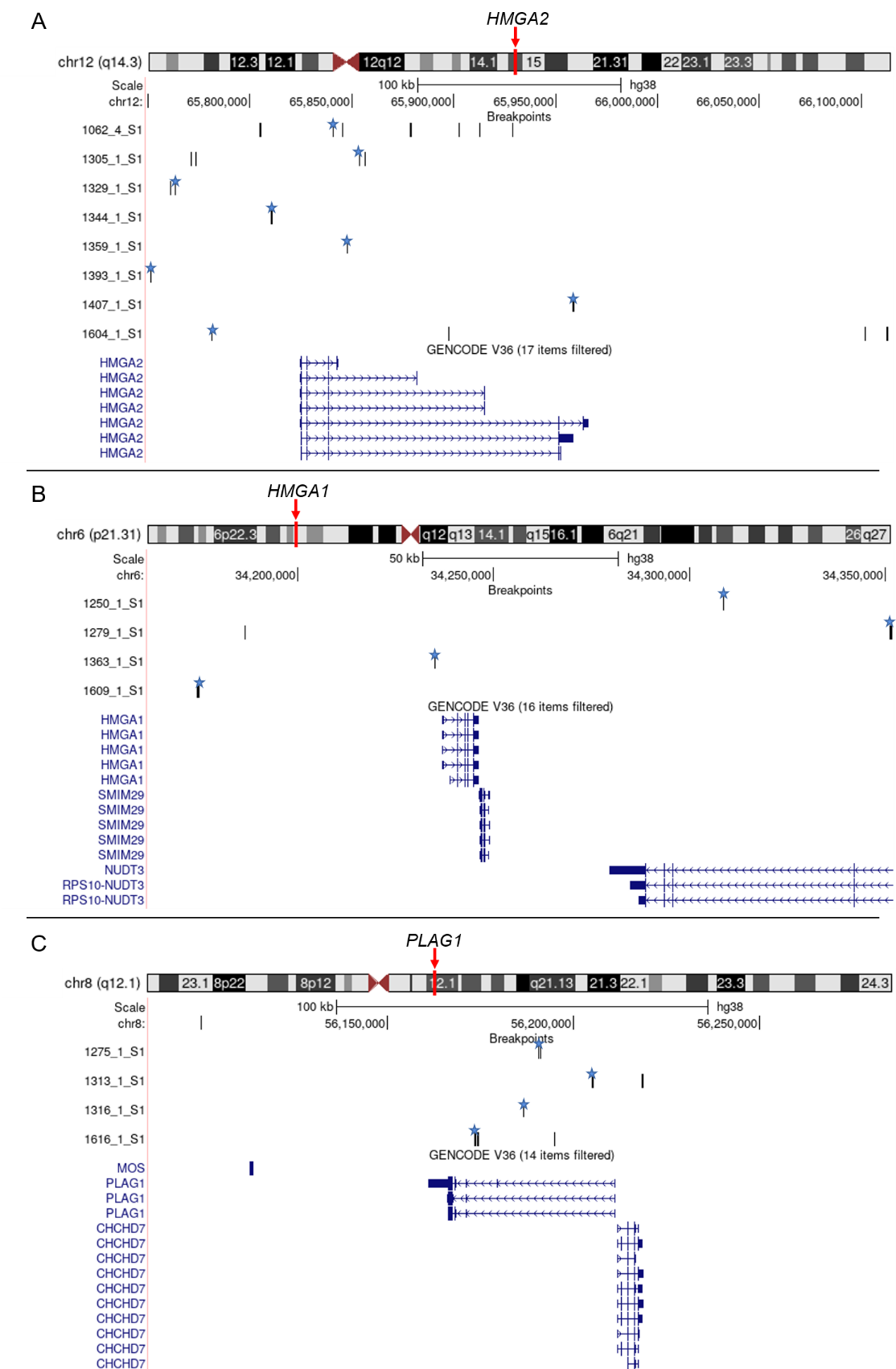


**Supplementary Figure 3. Breakpoints within, upstream, and downstream of *HMGA2*, *HMGA1*, and *PLAG1* identified by whole-genome sequencing of 16 leiomyomas.** Locations of breakpoints within, upstream, and downstream of **A)** *HMGA2,* **B)** *HMGA1,* and **C)** *PLAG1*. Breakpoints validated by Sanger sequencing are marked with a star. Intragenic breakpoints were detected in *HMGA2* and *PLAG1*, whereas chromosomal rearrangements targeting *HMGA1* included only upstream and downstream breakpoints. The figures were created using UCSC Genome Browser on Human Dec. 2013 (GRCh38/hg38) (https://genome.ucsc.edu/cgi.bin/hg/Gateway).


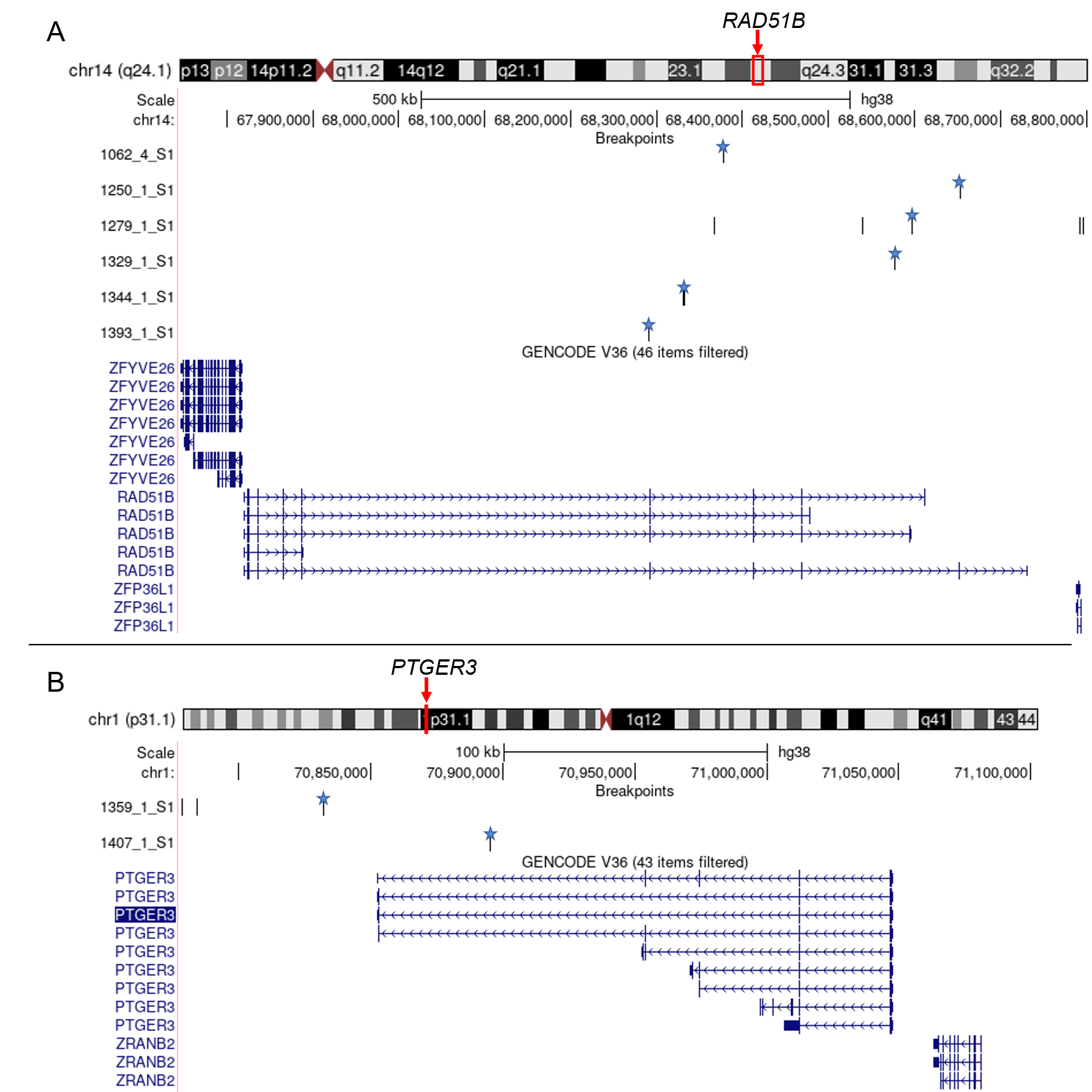
**Supplementary Figure 4. Breakpoints within, upstream, and downstream of recurrent partner genes identified by whole-genome sequencing of 16 leiomyomas.** **A)** We identified *RAD51B* as a putative translocation partner for *HMGA1* in two samples and *HMGA2* in four samples. Alterations included both simple translocations and complex rearrangements. **B)** We identified a region downstream of *PTGER3* as a recurrent translocation partner for *HMGA2* in two uterine leiomyomas. Breakpoints validated by Sanger sequencing are marked with a star. UCSC Genome Browser on Human Dec. 2013 (GRCh38/hg38) was used to create the figures (https://genome.ucsc.edu/cgi.bin/hg/Gateway).


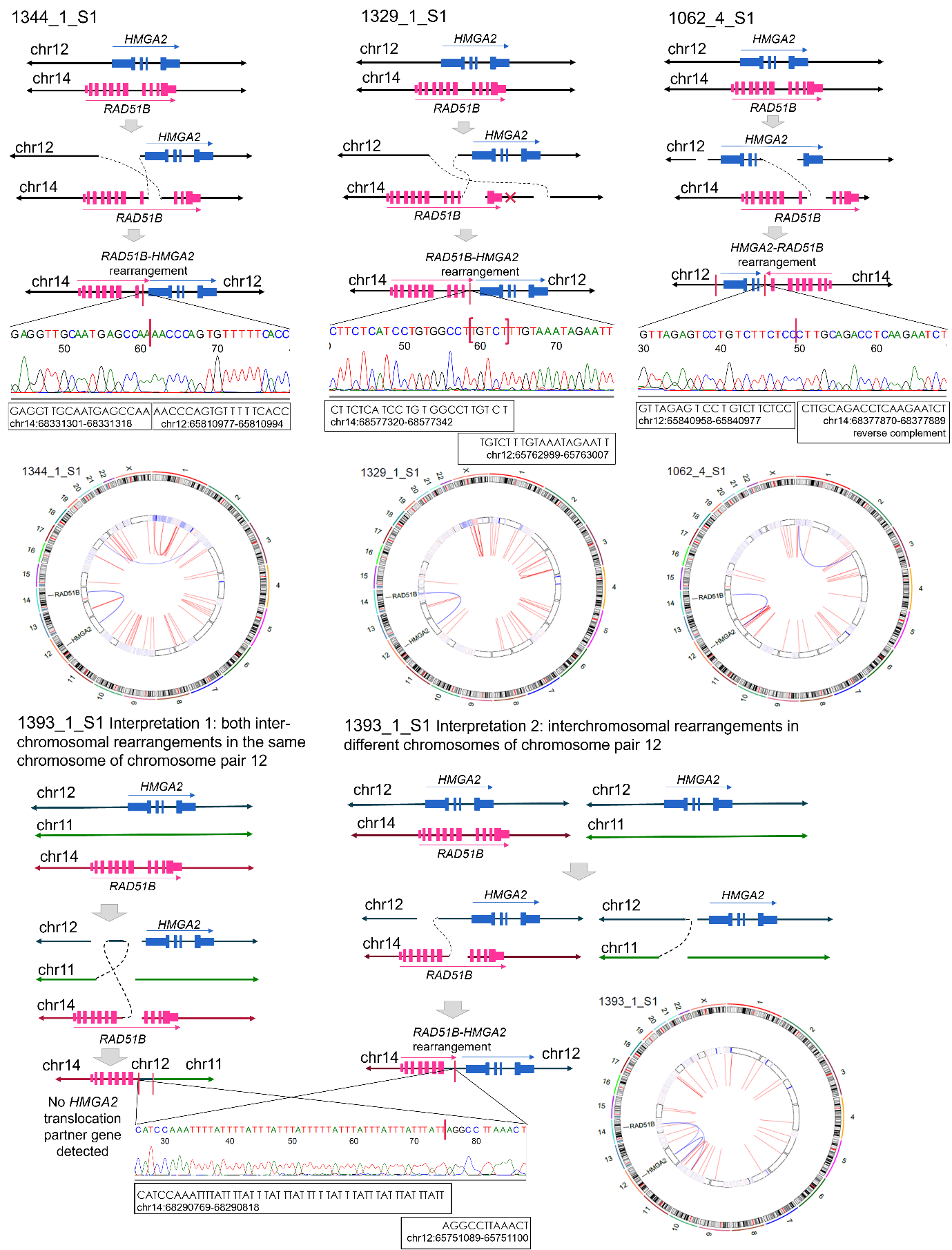


**Supplementary Figure 5. *HMGA2* rearrangements involving *RAD51B* in four leiomyomas.** In circos plots, intrachromosomal rearrangements are shown with red lines, interchromosomal rearrangements with blue lines, and copy number data by heatmap of the inner circle. Schematic figures are not in scale, and only relevant breakpoints are shown. Circos plots were made using RCircos v1.2.1 and Sanger sequences using FinchTV v1.4.0.^2,3^


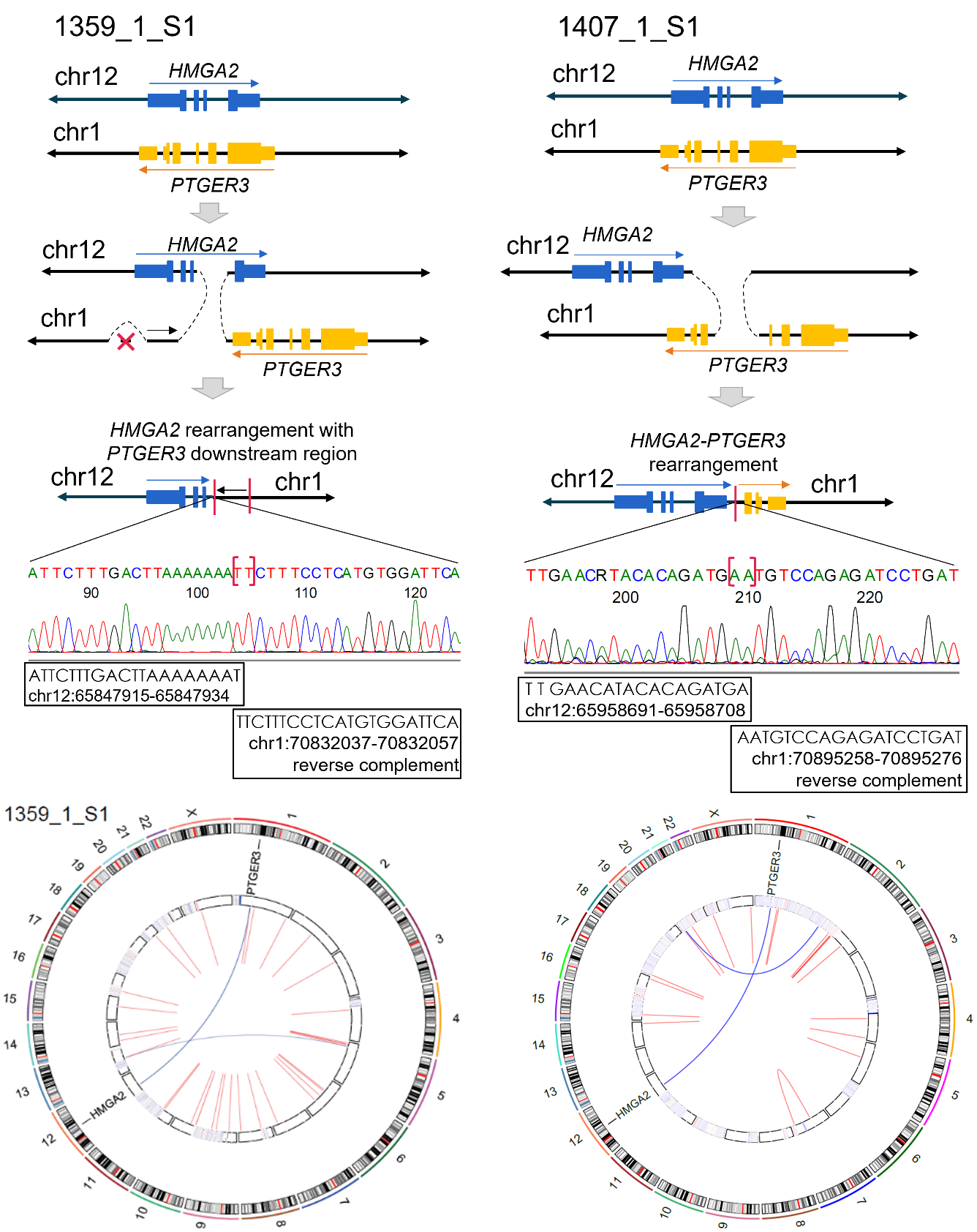


**Supplementary Figure 6. *HMGA2* rearrangements involving *PTGER3*.** We identified a region downstream of *PTGER3* as a recurrent candidate translocation partner in two leiomyomas. In the circos plots, intrachromosomal rearrangements are shown with red lines, interchromosomal rearrangements with blue lines, and copy number data by heatmap of the inner circle. Schematic figures are not in scale, and only relevant breakpoints are shown. Circos plots were made using RCircos v1.2.1 and Sanger sequences using FinchTV v1.4.0.^2,3^


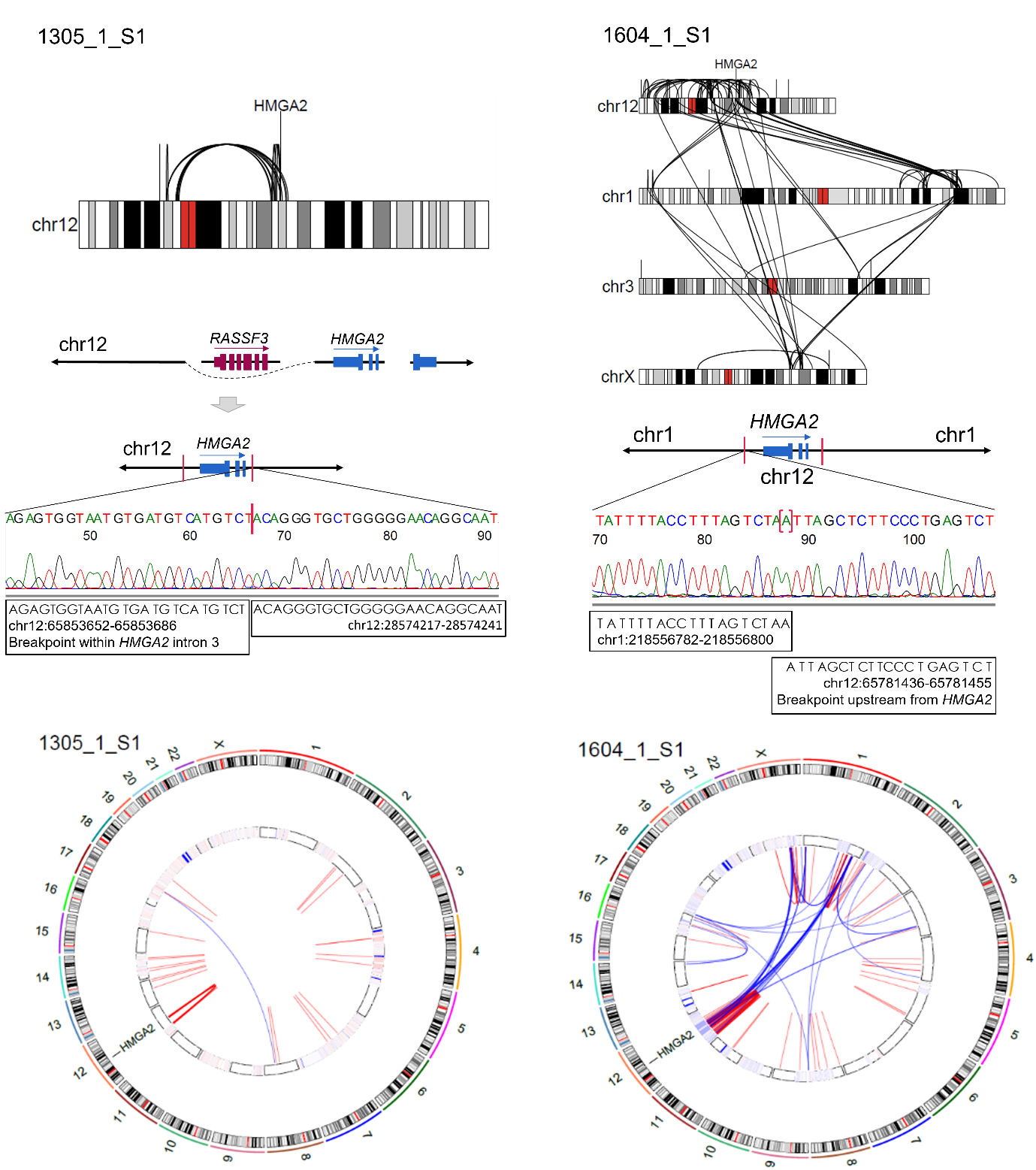


**Supplementary Figure 7. *HMGA2* rearrangements without a candidate partner gene.** We identified no partner gene for *HMGA2* in two leiomyomas. In the circos plots, intrachromosomal rearrangements are shown with red lines, interchromosomal rearrangements with blue lines, and copy number data by heatmap of the inner circle. Schematic figures are not in scale, and only relevant breakpoints are shown. Circos plots were made using RCircos v1.2.1, Sanger sequences using FinchTV v1.4.0, and idiograms with rearrangements using karyoploteR v1.18.0.^2-4^


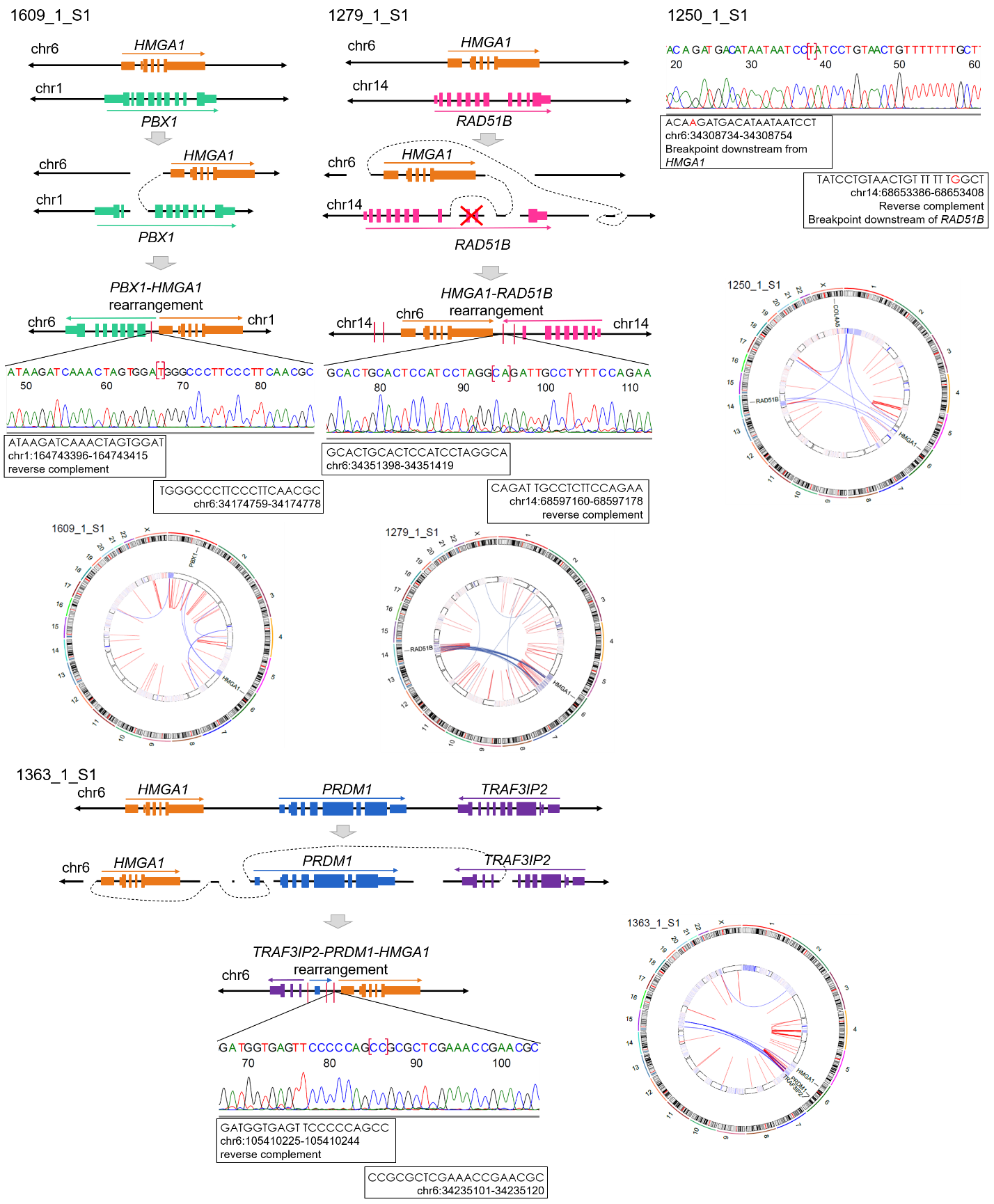


**Supplementary Figure 8. *HMGA1* rearrangements in four leiomyoma samples.** In sample 1609_1_S1, *HMGA1* was combined with the 3’end of *PBX1*. Two samples (1279_1_S1 and 1250_1_S1) displayed *HMGA1* rearrangements with the breakpoints within or close to *RAD51B*. Sample 1363_1_S1 harbored complex rearrangements, combining *HMGA1* with the 3’end of *TRAF3IP2* with a small fragment of *PRDM1* in between. In circos plots, intrachromosomal rearrangements are shown with red lines, interchromosomal rearrangements with blue lines, and copy number data by heatmap of the inner circle. Schematic figures are not in scale, and only relevant breakpoints are shown. Circos plots were made using RCircos v1.2.1 and Sanger sequences using FinchTV v1.4.0.^2,3^


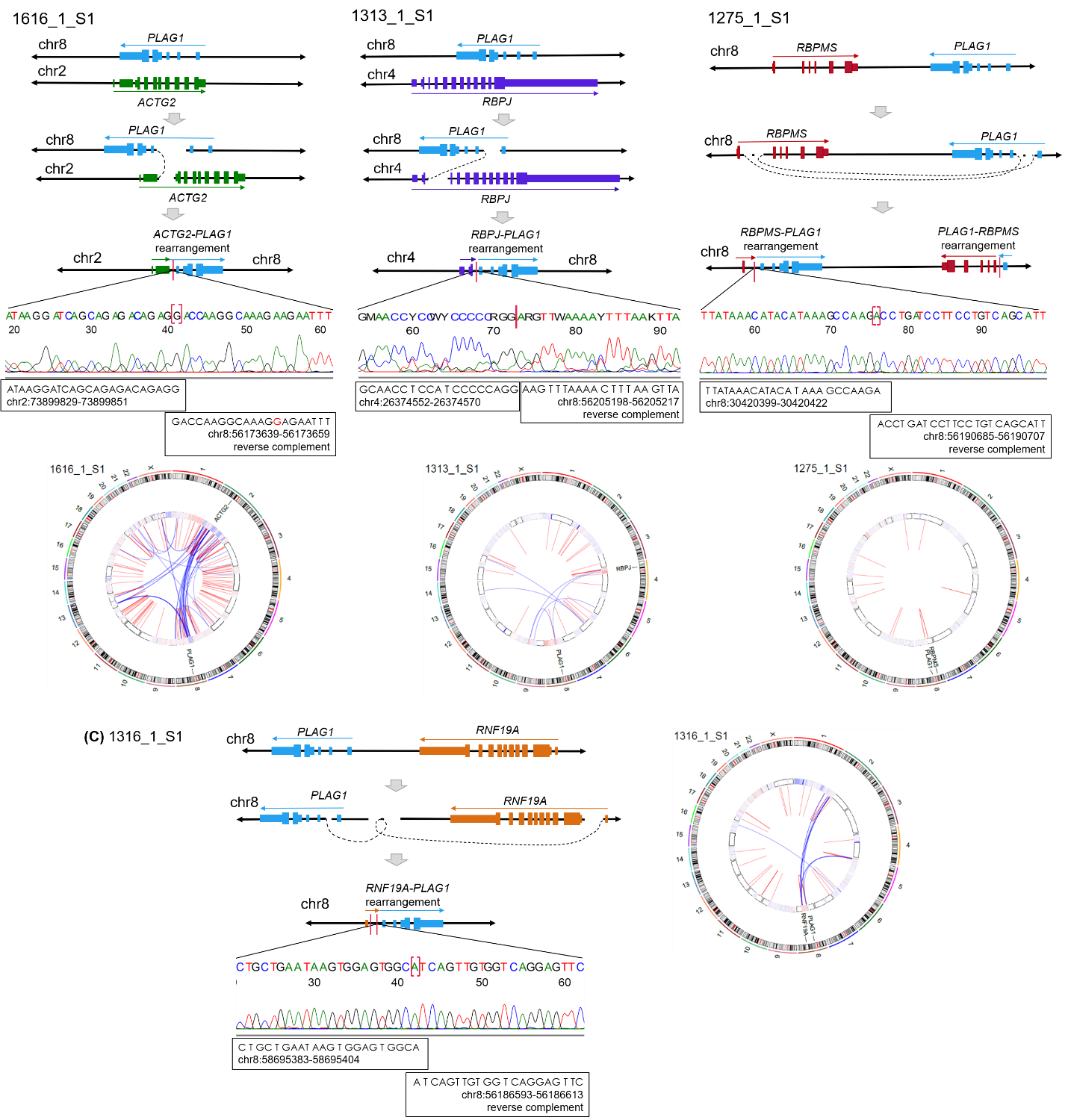


**Supplementary Figure 9. *PLAG1* rearrangements in four leiomyomas.** In each *PLAG1* rearrangement, the 3’ end of *PLAG1*, including all the coding exons, was combined with the 5’ end of another gene. The rearrangement partner genes (*ACTG2, RBPJ, RBPMS,* and *RNF19A*) are all highly expressed in the uterus in normal conditions, suggesting that their promoters and/or enhancers upregulate *PLAG1* expression in these rearrangements. In circos plots, intrachromosomal rearrangements are shown with red lines, interchromosomal rearrangements with blue lines, and copy number data by heatmap of the inner circle. Schematic figures are not in scale, and only relevant breakpoints are shown. Circos plots were made using RCircos v1.2.1 and Sanger sequences using FinchTV v1.4.0.^2,3^

**REFERENCES**

1. Talevich E, Shain AH, Botton T, Bastian BC. CNVkit: Genome-Wide Copy Number Detection and Visualization from Targeted DNA Sequencing. *PLoS Comput Biol.* 2016;12(4):1-18.
2. Zhang H, Meltzer P, Davis S. RCircos: An R package for Circos 2D track plots. *BMC Bioinformatics*. 2013;14(1).
3. FinchTV 1.4.0 (Geospiza, Inc.; Seattle, WA, USA; http://www.geospiza.com).
4. Gel B, Serra E. karyoploteR: an R/Bioconductor package to plot customizable genomes displaying arbitrary data. *Bioinformatics*. 2017;33(19):3088-3090.
